# Conformational Space Profiling Enhances Generic Molecular Representation for AI‐Powered Ligand‐Based Drug Discovery

**DOI:** 10.1002/advs.202403998

**Published:** 2024-08-29

**Authors:** Lin Wang, Shihang Wang, Hao Yang, Shiwei Li, Xinyu Wang, Yongqi Zhou, Siyuan Tian, Lu Liu, Fang Bai

**Affiliations:** ^1^ Shanghai Institute for Advanced Immunochemical Studies and School of Life Science and Technology Shanghai Tech University Shanghai 201210 China; ^2^ Shanghai Institute for Advanced Immunochemical Studies School of Life Science and Technology Information Science and Technology Shanghai Tech University Shanghai Clinical Research and Trial Center Shanghai 201210 China

**Keywords:** cellular‐level molecular activity modeling, conformational space, drug discovery, ligand‐based virtual screening, molecular representation learning

## Abstract

The molecular representation model is a neural network that converts molecular representations (SMILES, Graph) into feature vectors, and is an essential module applied across a wide range of artificial intelligence‐driven drug discovery scenarios. However, current molecular representation models rarely consider the three‐dimensional conformational space of molecules, losing sight of the dynamic nature of small molecules as well as the essence of molecular conformational space that covers the heterogeneity of molecule properties, such as the multi‐target mechanism of action, recognition of different biomolecules, dynamics in cytoplasm and membrane. In this study, a new model named GeminiMol is proposed to incorporate conformational space profiles into molecular representation learning, which extracts the feature of capturing the complicated interplay between the molecular structure and the conformational space. Although GeminiMol is pre‐trained on a relatively small‐scale molecular dataset (39290 molecules), it shows balanced and superior performance not only on 67 molecular properties predictions but also on 73 cellular activity predictions and 171 zero‐shot tasks (including virtual screening and target identification). By capturing the molecular conformational space profile, the strategy paves the way for rapid exploration of chemical space and facilitates changing paradigms for drug design.

## Graphical abstract

By incorporating the molecular conformational space profile into the contrastive pre‐training, the authors have developed a molecular representation model called GeminiMol, which is trained on a small‐scale molecular dataset but has a relatively strong generalization ability on large‐scale benchmark datasets. GeminiMol shows excellent performance on multiple downstream tasks, including ligand‐based virtual screening, target identification, and Quantitative Structure‐Activity Relationship (QSAR) modeling, etc., thus providing a useful molecular representation tool for artificial intelligence‐based drug design areas.

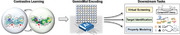

## 1 Introduction

If we say that representation learning is the “sensory receptor” of artificial intelligence (AI) for complex entities, as the modulator for transforming human‐readable information such as text, images, and sound into digital representations that can be understood by intelligent agents, then molecular representation learning is the interpreter to interpret the human‐readable molecular representations (SMILES and Graph) into feature vectors for AI models to learn to perform drug discovery tasks. Molecular representation learning enables us to train models to understand molecular representations through pre‐training tasks, thereby enhancing their performance in various downstream tasks. This approach is also known as meta‐learning. Over the past few years, the scientific community has devised three prominent training strategies for molecular representation learning, i.e., self‐supervised learning, supervised learning, and composite strategies.

Self‐supervised learning is a popular training strategy that trains models to understand different formats of molecular representations (SMILES, IUPAC, InChI, Graph, 3D structure, molecular fingerprints, and even molecular image), such as SMILES‐Bert,^[^ ^1^ ^]^ ChemBERTa,^[^ ^2^ ^]^ KPGT,^[^ ^3^ ^]^ NYAN,^[^ ^4^ ^]^ GraphMVP,^[^ ^5^ ^]^ GEM,^[^ ^6^ ^]^ Uni‐Mol,^[^ ^7^ ^]^ 3DGCL,^[^ ^8^ ^]^ ImageMol,^[^ ^9^ ^]^ etc. By leveraging contrastive learning or self‐generated on diverse molecular representations, self‐supervised pre‐trained models can effectively extract features of molecular structures without requiring molecular structure labels. Due to the requirement of learning millions of unlabeled molecular structures during the pre‐training stage, it is a challenge for us to assess the generalization capabilities of self‐supervised pre‐training in the chemical space that is far from the training data. In terms of performance on downstream tasks, Sun et al. have pointed out that partial self‐supervised pre‐training strategies do not always show obvious advantages over methods without pre‐training.^[^ ^10^ ^]^

The supervised pre‐training strategy is training with structure‐property projection or structure‐label semantic matching to capture molecular organic or pharmacological profiles, such as ChemBERTa‐2,^[^ ^11^ ^]^ MolT5,^[^ ^12^ ^]^ CLOOME,^[^ ^13^ ^]^ etc. For supervised pre‐training strategies, different types of molecular properties are required to form pre‐training tasks. These can be specific classification or regression tasks, but also retrieval or generation tasks of molecular descriptors or related biomedical data. The requirement of labeled data is the major drawback of supervised pre‐training strategies. Besides, the inclusion of molecular feature information in the pre‐training process raises concerns about the generalization ability of the model.

Considering the advantages and disadvantages of self‐supervised and supervised strategies, several composite strategies have been proposed to further improve the learning of molecular representation, such as HelixADMET.^[^ ^14^ ^]^ These strategies employ segmented pre‐training stages, which involve additional supervised pre‐training after self‐supervised pre‐training. At the self‐supervised stage, they include tasks such as self‐generation, molecular fingerprint generation, and 3D geometry prediction. At the supervised pre‐training stage, they include tasks such as molecular properties prediction. This composite strategy enables such models to achieve superior performance in most downstream tasks. However, the integration of self‐supervised and supervised pre‐training also has its weaknesses, such as inadequate chemical space representation in self‐supervised pre‐training, strong dependence on data quality and quantity in supervised pre‐training, and relatively poor generalization in downstream tasks.

To address the concerns about possible shortcomings in chemical space characterization and generalization capabilities in representation learning, we propose a hybrid contrastive learning framework, that performs inter‐molecular contrastive learning using inter‐molecular similarity as projection heads and enables training reliable molecular representation models without including experimental molecular properties. Using this strategy, this work presents a molecular representation model named GeminiMol that incorporates the full molecular conformational space, and pharmacophoric profiles by learning the Conformational Space Similarity (CSS) of drug molecules. The overall workflow of this study is illustrated in Figure S1 (Supporting Information). First, we defined descriptors for conformational space similarity. We then created CSS descriptors for a small dataset (39290 molecules) to train GeminiMol. Finally, we investigated the performance of our GeminiMol model in four important drug discovery tasks: virtual screening, target identification, Quantitative Structure‐Activity Relationship (QSAR), and prediction of Adsorption, Distribution, Metabolism, Excretion, and Toxicity (ADMET) properties prediction. We have extensively tested the GeminiMol model to evaluate its generalization performance in various drug discovery scenarios, thereby extending and deepening the research paradigm in the field of molecular representation learning.

## 2 Results

### 2.1 Descriptors of Conformational Space Similarity

The similarity between pairs of molecules provides an opportunity for contrastive learning. However, previous studies have used pairs of molecules that were generated by modification of the molecular structure, such as MolCLR.^[^ ^15^ ^]^ and iMolCLR,^[^ ^16^ ^]^ rather than similarities between pairs of molecules that are of pharmacological or physical significance. To further enhance the facilitative effect of contrastive learning on molecular representation learning, it is imperative to incorporate molecular similarity evaluation metrics that possess pharmacological or physical significance into the realm of molecular representation learning, such as 3D shape and pharmacophore similarity. Recently, MolCLaSS.^[^ ^17^ ^]^ incorporated the 3D shape similarity into the framework of contrastive learning. However, since the bioactive conformational space of drug molecules, plays an essential role in drug‐target binding,^[^ ^18^, ^19^ ^]^ a few minimal conformers used in previous works (GEM,^[^ ^6^ ^]^ Uni‐Mol,^[^ ^7^ ^]^ and MolCLaSS.^[^ ^17^ ^]^) cannot represent the bioactive conformational space corresponding to the drug‐target binding. To overcome the shortcomings of existing methods that do not fully represent the bioactive conformational space of molecules, this study uses a systematic conformational search method to enumerate all possible 3D conformations for each molecule to represent the molecule conformational space. Then the similarity between two molecules is evaluated between their 3D shape similarities of generated conformational space.

Most small molecules contain multiple rotatable bonds, and rotational arrangements produce different conformations. Theoretically, conformers generated by systematic conformational searching via rotating each bond with a small bin of angles can represent almost the entire conformational space of a molecule. A small molecule may adapt to different conformations while interacting with different binding pockets of proteins, e.g., imatinib, which binds to four different target proteins with three different conformations (as shown in Figure S2a, Supporting Information). This example indicates that conformational information is important to appropriately describe properties of drugs in drug action. As the number of rotatable bonds increases, the conformational space of a small molecule becomes more complicated, e.g., with more relatively low‐energetic conformational clusters (as shown in Figure S2b, Supporting Information), indicating more alternatives for recognizing different drug targets. As illustrated in Figure S3 (Supporting Information), the distribution of the computationally estimated strain energies of 6670 experimentally determined native conformations of 4518 molecules obtained from the protein data bank (PDB).^[^ ^20^ ^]^ indicates that the number of rotatable bonds is a decisive factor for the complexity of the conformational space of a molecule. Therefore, it is necessary to develop descriptors that can characterize the CSS for contrastive learning to describe a molecule more appropriately.

The main principle of the GeminiMol encoder is the extraction of conformational space features based on the 2D molecular structures. To achieve this goal, we monitor the acquisition of conformational space features by using different CSS descriptors (the details were described in the method section). To this end, we created a molecular dataset with a size of 39290 molecules and entirely scanned their conformational space. On this basis, two conformational ensembles were created for each molecule, one consists of near‐native conformers whose strain energies are lower than a defined threshold (0.5060 kcal mol^−1^ per rotatable bond), and the other is a larger conformation ensemble that has a higher strain energy threshold of 1.4804 kcal mol^−1^ per rotatable bond. The raw CSS descriptors (maximum and minimum similarity scores) under per threshold between pairs of molecules through pharmacophore and geometric shape alignment were generated by using PhaseShape.^[^ ^21^ ^]^ (the details were described in the *method* section). Moreover, advanced CSS descriptors were designed to data enhancement, which was obtained by numerical transformation to combine the raw CSS descriptors, i.e., MaxSim for portraying maximum similarity, MaxDistance for portraying maximum difference, MaxAggregation for portraying the closeness between conformational spaces, and MaxOverlap for portraying the degree of overlap between conformational spaces (the details were described in the *method* section).

### 2.2 Training and Interpreting the GeminiMol Framework

**Figure 1:**
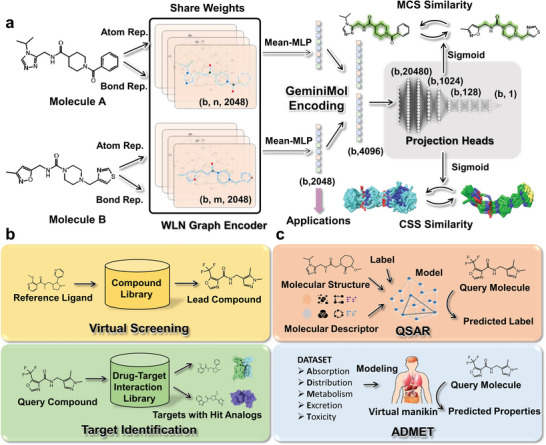
The contrastive learning framework to learn the conformational space profile and application tasks. a) The pre‐training and the application framework of GeminiMol. In the pre‐training stage, both MCS and CSS molecular similarities were considered in our model, the CSS descriptors were evaluated by PhaseShape (the details were described in the *method* section), and the MCS similarity was calculated by the MCS alignment. Molecules are fed into the network as graphs and encoded independently, where atoms are encoded as nodes and bonds are encoded as edges. Straight Arrows show the information flow among the various network components described in this paper. The highlighted 2D structures represent maximal topological common substructures (ignoring elemental differences). The dimensions of the feature vectors between the different network layers are labeled in parentheses. b) The zero‐shot application tasks of GeminiMol. By comparing the similarity between representation vectors or predicting the MCS or CSS similarities between molecules through the projection head, we can apply GeminiMol to molecular similarity prediction. c) The molecular property modeling tasks of GeminiMol. By training a decoder or fine‐tuning the GeminiMol model using representation vectors, we can establish GeminiMol‐based predictors for downstream tasks in molecular property prediction.

As an inter‐molecular contrastive learning framework, GeminiMol independently encodes the pair of molecules with the same encoder in the training stage (as illustrated in **Figure** **1**a). The raw molecular graph is updated by message passing of Weisfeiler‐Lehman Network (WLN).^[^ ^22^ ^]^ The molecular representations are read form the updated molecular graph. Next, the molecular representations are concatenated together and then fed into different projection heads to predict various CSS descriptors and Maximum Common Substructure (MCS) similarities (as shown in Figure 1a), the main purpose of these projection heads is to supervise the encoders in learning an informative molecular representation (named GeminiMol encoding). In contrast to the one or two hidden layers typically found in other contrastive learning frameworks,^[^ ^23^ ^]^ our projection heads contain a rectifier structure composed of multiple linear layers and activation functions (as shown in Figure 1a), designed to condense input features.

In addition to utilizing the inter‐molecular similarity descriptors as the contrastive learning objective to train independent molecular encoders, we also considered that the molecular similarity problem is similar to a sentence similarity problem in natural language processing. Therefore, we employed a chemical language‐based end‐to‐end molecular similarity prediction framework as a baseline model in this study, which is named CrossEncoder. The CrossEncoder allowed the informational communication between the reference molecule and the query molecule. It was implemented by adding a chemical tokenizer to ELECTRA.^[^ ^24^ ^]^ model using AutoGluon.^[^ ^25^ ^]^

To evaluate the GeminiMol, CrossEncoder, molecular fingerprints, and other third‐party baseline methods, we applied them to various downstream benchmark tasks as shown in Figure 1b,c. Benchmark datasets and corresponding baseline methods for these different downstream tasks are created: 1) the DUD‐E.^[^ ^26^ ^]^ and LIT‐PCBA.^[^ ^27^ ^]^ for virtual screening; 2) Target Identification Benchmark Dataset (TIBD) for target identification; 3) The target‐level and cellular‐level compound activity data for QSAR; and 4) ADMET‐related compound properties data for ADMET modeling. Further information can be found in the *method* section for details. These four categories of tasks encompass the main scenarios required for ligand‐based drug design, providing a comprehensive assessment of the overall performance of different molecular representation methods in ligand‐based drug design tasks. Compared to previous studies, we introduced the target identification in zero‐shot learning tasks (as shown in Figure 1b), and incorporated cellular‐level activity prediction into molecular property modeling tasks.

**Figure 2:**
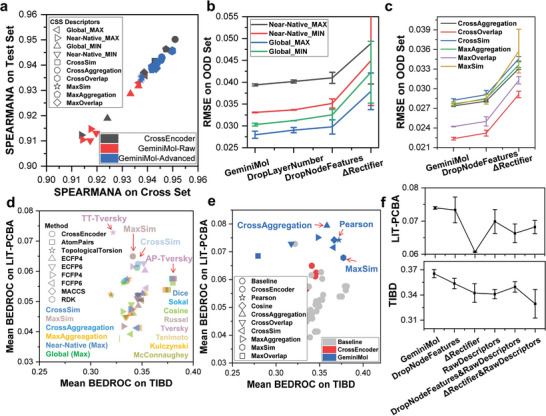
Testing and interpreting the GeminiMol models on CSS predictions and zero‐shot drug discovery tasks. a) The correlation of performance between the cross and test sets reveals the high generalization capability of our models. The various CSS descriptors were represented by different shapes. For instance, Global_MAX represents the maximum similarity in the global conformational space, and so on. Among them, MaxSim represents the maximum value of all similarities, MaxDistance represents the minimum similarity, MaxOverlap represents one‐half of the sum of MaxSim and MaxDistance, and MaxAggregation represents one‐half of the sum of the maximum similarities under both strain energy thresholds. If “Max” is replaced by “Cross” in the names of the similarities in this work, this means half the value of the sum of the similarities of the molecules A to B and B to A. b) Ablation results of raw CSS labels. c) Ablation results of transformed CSS descriptors. The GeminiMol model was subjected to four repeated training and testing experiments, while the other models underwent a minimum of two repeated experiments. The term DropLayerNumber refers to reducing the number of layers in the WLN network to 2. The term DropNodeFeatures indicates the reduction of node dimensionality in the WLN network to 1024. The term ΔReacifier denotes replacing the projection head to a simplification deep neural network. d) The performance comparison between CrossEncoder with molecular fingerprints. The virtual screening performance is indicated by the Boltzmann‐enhanced discrimination of receiver operating characteristic (BEDROC) metric in the LIT‐PCBA benchmark test, and the target identification performance is also BEDROC in the TIBD benchmark test. The choice of LIT‐PCBA over DUD‐E is primarily because the molecules in LIT‐PCBA are derived from real high‐throughput screening datasets. The model with the best balanced performance was pointed out with a red arrow. Molecular presentation methods and fingerprints were represented by various shapes, while similarity metric indices were depicted using different colors. The various CSS descriptors were represented by different colors. e) The performance comparison of different molecular fingerprints, CrossEncoder, and GeminiMol. The various CSS descriptors were represented by different shapes. The similarity calculated by the baseline method was represented by gray circles. The Pearson or Cosine mean similarities of GeminiMol encoding vector. f) Ablation results of GeminiMol models and CSS descriptors. The Pearson similarity metric was used in these ablation experiments for zero‐shot drug discovery. The GeminiMol model was subjected to four repeated training and testing sessions, while the other models underwent a minimum of two repeated experimental and testing sessions. The term DropLayerNumber refers to reducing the number of layers in the WLN network to 2. The term DropNodeFeatures indicates the reduction of node dimensionality in the WLN network to 1024. The term ΔReacifier denotes replacing the projection head to a simplification deep neural network. The term RawDescriptors represents the usage of raw CSS descriptors instead of advanced CSS descriptors for contrastive learning during the training process.

The CrossEncoder and GeminiMol were trained using a total of 8 million molecular similarity descriptors obtained through sampling pairs of molecules from 37336 molecules in our molecular dataset, and then evaluated on both the cross and test sets. It is important to note that the pairs of molecules in the cross set contain one molecule from the training set, whereas the pairs of molecules in the test set do not have any molecules from the training set. As shown in **Figure** **2**a, the comparison of the Spearman correlation coefficients of the models on the test and cross sets showed a strong correlation, indicating their robust generalization ability. According to our analysis of a subset with a size of 600 000, as shown in Figure S4 (Supporting Information), the pairs of molecules that are significantly different in 2D structures but have high similarity in 3D structures are very few, and can be counted as high‐quality out‐of‐distribution (OOD) samples. OOD testing is increasingly popular for evaluating a machine learning system's ability to generalize beyond the biases of a training set.^[^ ^28^ ^]^ Hence, based on these data, we performed ablation experiments to investigate the learning ability of each component in the GeminiMol model. As illustrated in Figure 2b, reducing the number of layers and the dimensionality of node features, as well as removing rectifiers in the projection heads, all have a detrimental effect on the model's performance. Similarly, the same trend can be observed for advanced CSS descriptors, as depicted in Figure 2c.

### 2.3 Toward Zero‐Shot Learning for Drug Discovery with GeminiMol Encoding

In the field of ligand‐based drug design methods, the principle is that molecules that have similar structures also have similar biological activities. This allows us to predict the similarity of biological activities between molecules by comparing the similarity of ligands, i.e., molecules that exhibit structural similarity to the reference molecule may possess similar biological activities or targets as the reference molecule. This enables the application of ligand similarity in virtual screening and target identification. In the field of machine learning, this strategy is also referred to as zero‐shot learning.^[^ ^29^ ^]^ Hence, molecular similarity searching serves as the primary methodology employed in zero‐shot learning for drug discovery.

To predict the molecular similarity using GeminiMol, a straightforward approach is to use projection heads to predict the CSS descriptors between molecules. Another approach is the direct comparison of the vector similarity between GeminiMol encodings, such as cosine similarity. It is worth noting that in this study, our molecular dataset (39290 molecules) has few overlaps with DUD‐E.^[^ ^26^ ^]^ (1463336) and LIT‐PCBA.^[^ ^27^ ^]^ (2808885). Therefore, the model needs to learn the relationship between the 2D molecular structures and their CSS while having excellent generalization ability to achieve better performance on downstream tasks.

PhaseShape.^[^ ^21^ ^]^ is a 3D pharmacophore and shape alignment method that was used in this study for scoring the similarity between each pair of conformers. Therefore, we initially compared the performance of the CrossEncoder and GeminiMol with PhaseShape.^[^ ^21^ ^]^ on the virtual screening to investigate whether the incorporation of conformational space profile is useful for virtual screening. As shown in Figure S5 (Supporting Information), many of the CSS descriptors and vector similarity of GeminiMol encodings, have significantly better performance than PhaseShape on the benchmark datasets of DUD‐E.^[^ ^26^ ^]^ and LIT‐PCBA.^[^ ^27^ ^]^ Particularly, the Pearson and cosine similarity between GeminiMol encodings exhibited superior performance in both virtual screening benchmarks.

To assess the differences between our models and prominent baseline methods, we evaluated the performance of 8 popular molecular fingerprints, CrossEncoder, and GeminiMol on the LIT‐PCBA.^[^ ^27^ ^]^ and the TIBD. As the baseline method, molecular fingerprints are considered the prevailing approach for compound 2D structural similarity search.^[^ ^30^ ^]^ We compared the molecular fingerprints with the CrossEncoder (as shown in Figure 2d) and GeminiMol (as shown in Figure 2e). The CrossEncoder demonstrated comparable performance to molecular fingerprints, mainly attributed to the MaxSim and CrossSim predictors. However, its performance did not surpass that of molecular fingerprints. Surprisingly, the cosine similarity and Pearson correlation of the GeminiMol encoding exhibited balanced and superior performance across virtual screening and target identification compared to other methods. Among the various GeminiMol similarities, the Pearson correlation coefficient achieved the best overall performance on virtual screening and target identification. Further ablation experiments (as shown in Figure 2f) demonstrate that removing the rectifier in the projection head significantly affects the performance and robustness of the GeminiMol, consistent with previous ablation experiment findings. Additionally, reducing the features of the node and substituting advanced descriptors with raw CSS descriptors both resulted in performance degradation of the model in virtual screening and target identification tasks.

### 2.4 Applying GeminiMol Encoding for Identifying new Bioactive Molecules

To meet the requirement of compound novelty in drug discovery, the molecular representation model should effectively capture the underlying similarities between these molecules, even in cases where there are notable differences in their 2D structures. This aspect is of great importance in practice, especially in the identification of new active molecules with different scaffold structures. Compared to baseline methods, GeminiMol has demonstrated exceptional performance in zero‐shot learning, such as virtual screening and target identification (as illustrated in Figure 2e). To gain insights into the underlying factors contributing to GeminiMol's good performance, we investigated its sensitivity to structural changes and scaffold hopping of drugs for three different targets.

**Figure 3:**
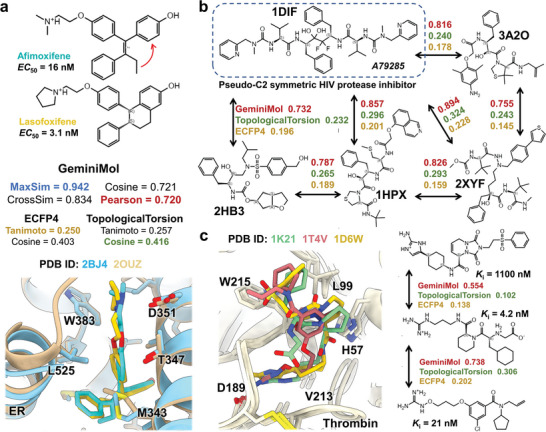
Applying the GeminiMol encoding on the molecular similarity evaluation. a) Changes in molecular similarity induced by the type of chemical modification of ring‐closure. After the structural changes, significant variations in molecular fingerprint similarity were observed, with all metrics below 0.5. However, GeminiMol similarity still maintained a score of 0.7 or higher. b) Intrinsic similarities of symmetric and non‐symmetric HIV protease inhibitors. The molecular fingerprints were unable to identify the similarity between these molecules with different scaffolds (with scores below 0.4). However, GeminiMol considered the similarity scores for these molecules to be above 0.7. The Pearson similarity metric was used. c) For three molecules with significant differences in their 2D structures but exhibit similar binding modes within protein pockets, the Pearson similarity of GeminiMol encoding is able to recognize their intrinsic similarity. For instance, the weaker binder has a similarity score of 0.554, while the stronger binder has a score of 0.738.

We undertook a preliminary analysis to evaluate the impact of common ring‐closure operations on the refinement of lead compounds, employing GeminiMol encoding, ECFP4,^[^ ^31^ ^]^ and TopologicalTorsion^[^ ^32^ ^]^ fingerprints, as depicted in **Figure** **3**a. We focused on two non‐steroidal estrogen receptor modulators, namely lasofoxifene and afimoxifene. Both compounds act identically on the estrogen receptor which are structurally analogous, exhibiting comparable binding modes.^[^ ^33^, ^34^ ^]^ (refer to Figure 3a), and their similarity remain within the predictive power of visual inspection. However, the molecular fingerprints indicate considerable structural differences. In contrast, GeminiMol, which recognizes these nuanced structural changes as distinct analogs, predicted a MaxSim value of 0.942, associated with an encoding similarity of over 0.7.

The HIV protease has been a significant target in drug development with a comprehensive research history. Initially, research predominantly focused on pseudo‐symmetric peptide inhibitors, evolving to diverse chemical scaffolds, while structurally different from the original peptide inhibitors, share a consistent mechanism of action. A comparative analysis of the structural diversity of these inhibitors was performed, starting with the classical pseudo‐symmetric inhibitor A79285.^[^ ^35^ ^]^ against four non‐symmetric inhibitors.^[^ ^36^, ^37^, ^38^ ^]^ The molecular fingerprints suggested the 2D structural diversity of these molecules, but it was also found that these molecules are close to each other in the encoded space of GeminiMol (as shown in Figure 3b). This observation highlights GeminiMol's effectiveness in detecting intrinsic similarities within the conformational space of diverse molecules.

In a similar vein, we conducted a comparative analysis of the molecular structures of three thrombin inhibitors and their respective interaction patterns with thrombin.^[^ ^39^, ^40^, ^41^ ^]^ This study demonstrates that the GeminiMol Model is able to identify fundamental similarities between these molecules despite significant differences in their 2D structures. In particular, we observed a correlation between the activity of the inhibitors and their similarity to the most potent inhibitor, whereby lower similarity is associated with lower activity (as shown in Figure 3c).

### 2.5 Evaluating GeminiMol Encoding on the Molecular Property Modeling

Molecular property modeling is a widely used approach for evaluating the performance of molecular representation models in downstream tasks. In previous studies, datasets containing ADMET property information and target‐level biological activity data, such as MoleculeNet,^[^ ^42^ ^]^ Therapeutics Data Commons (TDC),^[^ ^43^ ^]^ LIT‐PCBA,^[^ ^27^ ^]^ and PubChem BioAssays,^[^ ^44^ ^]^ have been utilized to assess the representational capabilities of various molecular representation methods. In this study, to comprehensively evaluate the performance of various molecular representation methods, we collected 21 target‐level biological activity datasets from LIT‐PCBA and PubChem BioAssays,^[^ ^44^ ^]^ 73 cellular‐level biological activity datasets from National Cancer Institute Developmental Therapeutics Program (NCI/DTP),^[^ ^45^ ^]^ 46 ADMET property datasets from TDC,^[^ ^43^ ^]^ as well as 1 dataset on drug addiction.^[^ ^46^ ^]^ (refer to the *methods* section for details). In particular, QSAR tasks are divided into two categories: target‐level and cellular‐level. The former is associated with specific targets, while the latter may involve multiple unknown targets and cytotoxicity mechanisms.

**Figure 4:**
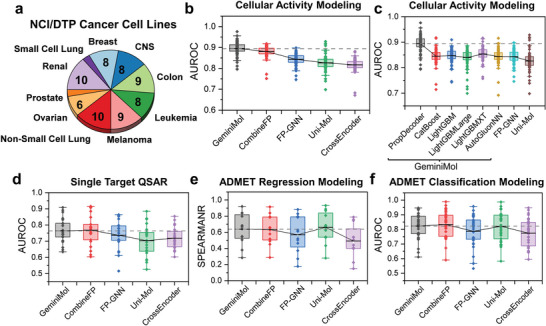
Applying the GeminiMol encoding on the molecular property modelings. a) statistics of 73 cancer cell lines in the NCI/DTP. b) the comparison between GeminiMol and other baseline methods in 73 cellular‐level biological activity modeling tasks. The dashed line indicates the performance of the GeminiMol model. c) The impact of decoder types on the performance of GeminiMol for cellular‐level biological activity modeling tasks. Among them, PropDecoder is a multilayer perceptron for fine‐tuning the GeminiMol model. CatBoost, LightGBM, and AutoGluonNN are prediction models constructed based on fixed encoders. The dashed line indicates the performance of the fine‐tuned GeminiMol‐PropDecoder model. d–f) illustrate the comparison between GeminiMol and other baseline methods in target‐level QSAR and ADMET property modeling tasks. These included 21 target‐level QSAR tasks, 35 classification, and 12 regression ADMET tasks. The dashed line indicates the performance of the GeminiMol model.

We compared the representation performance of GeminiMol and various molecular fingerprints on two different QSAR tasks, classification and regression ADMET tasks (see Figure S6, Supporting Information). The results show that CombineFP (combined by ECFP4,^[^ ^31^ ^]^ FCFP6,^[^ ^31^ ^]^ AtomPairs,^[^ ^47^ ^]^ and TopologicalTorsion.^[^ ^32^ ^]^) performs the best among all fingerprint methods. Due to the significant role of cellular‐level biological activity modeling for phenotype‐based drug discovery, we specifically focused on evaluating the performance of GeminiMol, molecular fingerprints, and third‐party baseline methods (including FP‐GNN and Uni‐Mol) in QSAR tasks across 73 different cancer cell lines (as shown in **Figure** **4**a). Among these molecular representation methods, GeminiMol model exhibit similar optimal performance, significantly outperforming CombineFP and other baseline methods (as shown in Figure 4b).

To elucidate the reasons behind the superior performance of GeminiMol in cellular‐level QSAR tasks, we conducted a comparison of different decoders and their impact on performance (as shown in Figure 4c). The results revealed that the PropDecoder, which emulates the projection head design in the GeminiMol framework, exhibited the best performance. As demonstrated in Figure S2 (Supporting Information), it can be observed that imatinib binds to different targets by multiple conformations. This implies that conformational space profile plays a significant role in modeling the multi‐target interactions and complex drug‐target network, thereby explaining the superior performance exhibited by GeminiMol in cellular‐level QSAR tasks. Besides, GeminiMol demonstrates a comparable performance with other state‐of‐the‐art methods in the target‐level QSAR and ADMET tasks (as shown in Figure 4d–f).

Furthermore, we investigated the performance of GeminiMol when only the original GeminiMol encoding is used as input features for downstream tasks while the encoder remains fixed. Under this configuration of a fixed encoder, GeminiMol exhibits a slight decline in performance. However, it still outperforms the majority of baseline models in QSAR tasks and surpasses FP‐GNN.^[^ ^48^ ^]^ and certain molecular fingerprints in ADMET tasks (as shown in Figure S7, Supporting Information). Remarkably, the fixed encoder was trained using only 37 336 molecular structures. This suggests that our training strategy endows the GeminiMol encoder with robust generalization capabilities. Additionally, incorporating a more diverse range of molecular structures in future research will further bolster the performance of GeminiMol.

Overall, the GeminiMol model is comparable not only to traditional molecular fingerprints but also FP‐GNN,^[^ ^48^ ^]^ which is specifically designed for QSAR tasks, and Uni‐Mol,^[^ ^7^ ^]^ which incorporates 19 M molecular structures during pre‐training, in terms of molecular presentation capability. This indicates that our proposed contrastive learning framework based on molecular conformational space similarity descriptors is an efficient strategy for training molecular representation models.

## 3 Conclusion

The data quantity and quality pose significant challenges in AI for science. This study introduces an innovative approach to tackle this predicament, by harnessing highly precise techniques from molecular simulation to yield training data of superior quality for AI. The primary objective of this approach is to mitigate the scarcity of high‐quality data in deep learning. By doing so, it facilitates the independent training of molecular representation models, while also deed as a supplementary data source for other strategies. Through our investigation, we have unearthed that incorporating descriptors of conformational space similarity can enhance the model's representative capacity and efficacy in drug discovery. In contrast to similar work, in designing our framework we have been careful not to include information that has already been included in other methods of representation, such as molecular fingerprints. Instead, we aim to explore novel means of representation, thereby paving the way for future model improvements.

Target‐based drug design has been the dominant paradigm in drug discovery in recent years.^[^ ^49^ ^]^ However, recent studies have shown that that most drugs have been discovered using phenotype‐based strategies at the cellular‐level and that some of the target‐based drugs also exert their effects through off‐target interactions.^[^ ^50^ ^]^ This finding suggests that there are still other important potential applications regarding to ligand‐based drug design area, including scaffold hopping and ligand similarity screening. Our work has highlighted room for improvement in current molecular representation models, inspiring us to drive a paradigm shift in drug discovery. With the implementation of initiatives such as Target 2035,^[^ ^51^ ^]^ which focuses on the development of compound probes, target identification based on ligand similarity is becoming increasingly important. Alongside advancements in molecular presentation techniques such as GeminiMol, the prediction of bioactivities and even the rational design of small molecules with specific target selectivity based on similarity networks between small molecules is becoming a new paradigm in future drug design. This study will also contribute to the fields of drug‐target interaction prediction and drug side effect prediction.

Note that, the conformational space profile is not a panacea for drug discovery. For a portion of tasks, the 2D structure of a compound already contains sufficient information to establish structure‐activity relationships, rendering the introduction of the conformational space profile inconsequential for these tasks. In recent years, driven by representative datasets of drug‐target 3D interactions, such as PDB‐Bind,^[^ ^52^, ^53^, ^54^, ^55^ ^]^ AI methods based on 3D interaction modeling between drugs and targets, such as PLANET,^[^ ^56^ ^]^ have been developed for drug‐target interaction prediction. Additionally, the properties of drug‐target binding require the inclusion of target information, such as binding affinity,^[^ ^57^, ^58^ ^]^ and binding kinetics.^[^ ^59^ ^]^ Besides, the absorption, distribution, and metabolism of many drugs are also closely related to the involvement of other biomacromolecules. Therefore, relying solely on information from the conformational space of small molecules to solve such problems is not an effective strategy. GeminiMol aims to improve existing small molecule representation methods and provide a practical tool for other downstream tasks, such as protein‐ligand affinity prediction and conditional molecule generation.

## 4 Experimental Section

### Strain Energy Analysis for Native Conformations Bound to Targets

Strain energy serves as an indicator of the energy difference between the ligand in its free state and its bound state. In our study, we analyzed the strain energy associated with the native conformations bound to targets in experimental structures to determine the threshold of strain energy in conformational searching. The 6672 native conformations obtained from the literature^[^ ^60^ ^]^ were first evaluated by static energy calculations using the Prime module of Schrödinger2021‐1, and we discarded 2 high‐energetically conformations (higher than 100 kcal mol^−1^) due to their implausible structures. Then, the strain energy analysis was performed over these conformations by the strain energy rescoring program of Schrödinger2021‐1. The solvent model used in the strain energy calculation procedure is the 4r distance‐dependent dielectric (4RDDD) model and the global conformational searching method used is MCMM/Low‐Mode in the MacroModel module of Schrödinger2021‐1.

It is important to determine an optimal factor for assigning appropriate strain energy thresholds to molecules of various sizes, which can be achieved by dividing the strain energy value by the number of rotatable bonds or heavy atoms presenting in the molecule (as shown in Figure S3, Supporting Information). The density plot demonstrates that rotatable bonds are the most representative factor, which is consistent with previous reports in the literature.^[^ ^60^, ^61^ ^]^

### Constructing the Small‐Scale Molecular Dataset

Considering the cost involved in sampling CSS descriptors as well as the diversity of the compound library, we constructed a much smaller dataset (39290 molecules in total, compared to 19 million in Uni‐Mol.^[^ ^7^ ^]^). As illustrated in Figure S8a (Supporting Information), this dataset consists of six different subsets: 1) the processed PDB ligands obtained from the literature.^[^ ^60^ ^]^ and contained 4461 unique molecules; 2) active ligands from the GPCR ligand library.^[^ ^62^ ^]^ contained 16513 unique molecules; 3) the active sets of Demanding Evaluation Kits for Objective In silico Screening.^[^ ^63^ ^]^ contained 2997 unique molecules; 4) DDS‐10 collection of Enamine diversity libraries (Enamine Ltd.) contained 10214 unique molecules; 5) Glide decoy molecules library (Schrödinger Inc.) contained 1920 unique molecules; and 6) the ChemBridge macrocyclic compounds library (ChemBridge Co.) contained 3156 unique molecules. The overlap between six different subsets is illustrated in Figure S8a (Supporting Information). We prepared them to generate protonation/tautomerization states with default settings by the LigPrep module of Schrödinger2021‐1, then, we generated the molecular SMILES representation by Schrödinger2021‐1.

We conducted an analysis of the molecular dataset to examine the distribution of molecular size and flexibility, as indicated by the number of heavy atoms and rotatable bonds, respectively. As shown in Figure S8b,c (Supporting Information), the distribution of molecular size and flexibility within a wide range, thereby providing potential diversity in the basic properties of the molecules in our molecular dataset. We further analyzed the structural diversity distribution of these compounds by Self‐Organizing Map (SOM), as shown in Figure S8d (Supporting Information). This map was created using 96 most informative bits of the pairwise fingerprint of Schrödinger packages, the color gradient within cell population was set to 114 – 1000, and the max population set to 1673. We demonstrated the relationship between the molecular structure and its SOM cell location in Figure S8d (Supporting Information), where compounds between neighboring cells have a similar structure. In the preparation of SOM, the molecular fingerprints were computed by pairwise fingerprint and the 96 more informative bits were used for the inference of SOM.

### Schemes for CSS Descriptors Collection

In this study, a workflow was proposed to estimate the CSS descriptors for the pairs of small molecules, as shown in Figure S9 (Supporting Information), i.e., conformational searching and 3D alignment. The CSS descriptors were calculated in three steps:

1. a pair of conformational ensembles were searched using a conformational searching method named MacroModel module in Schrödinger2021‐2. The maximum number of total steps was set to 2000, and the maximum number of per rotatable bond was 100. For each molecule, we aim to obtain a representative conformational space resembling near‐native conformers, as well as a conformational space that covers global conformers through systematic searching. Therefore, we have devised two schemes for conformational searching: i) global scheme: The Monte Carlo Multiple Minima (MCMM) method was employed for systematic conformational searching. For each molecule, the strain energy threshold was set as the number of rotatable bonds multiplied by 1.4806 kcal mol^−1^. The RMSD cutoff for conformational clustering was set at 1 Å. ii) near‐native scheme: The Low‐Mode/MCMM mixed method was employed for conformational searching. For each molecule, the strain energy threshold was set as the number of rotatable bonds multiplied by 0.5060 kcal mol^−1^. The RMSD cutoff for conformational clustering was set at 2 Å. In the strain energy analysis of the native conformers mentioned earlier, 0.5060 represents its first quartile, while 1.4806 represents its upper 95% CI of mean (as shown in Figure S3, Supporting Information).
2. the conformational ensemble of the query to reference molecule was superimposed using PhaseShape. The pharmacophore mode (‐pharm) of PhaseShape.^[^ ^21^ ^]^ was used for superimposing and scoring similarity between the conformers of the query molecule to the conformers of reference molecule (as shown in Figure S9a, Supporting Information).
3. the raw CSS descriptors were calculated by the maximum and minimum similarities of a pair of conformational ensembles, which represent the similarity of the conformational space for a pair of small molecules. Then, raw CSS descriptors were mapped into a matrix and transformed to advanced descriptors (as shown in Figure S9b, Supporting Information).

### Collecting the CSS Descriptors

We drew a random sample of 600 000 pairs of molecules and calculated the MCS and CSS with the conformers sampled under the different strain energy thresholds and RMSD thresholds as described above. The correlation between MCS similarity and the MaxSim was analyzed. The vast majority of pairs of molecules are concentrated in an elliptical region as shown in Figure S4 (Supporting Information), while the upper right corner represents the region with high MCS similarity and high CSS. We labeled these three regions as dense, sparse, and analogous, respectively. Given this unbalanced distribution, we increased sampling in the sparse and subsequently reduced sampling in the dense region.

As illustrated in Figure S9b (Supporting Information), all the pairs of molecules were categorized according to two dimensions: 1) training (37336), validation (598), test (1356), and crossover, and 2) dense, sparse, and analogous (as the ratio 0.30:0.65:0.05). The train, validation, and test sets were divided by molecular skeletons. For each pair, the indices of the two molecules were exchanged to obtain the value of the symmetric position in the matrix. For the model using SMILES as input, i.e., CrossEncoder, each SMILES in the dataset was randomized with a probability of 0.9 for data augmentation. We performed 10000 rounds of molecular sampling on the training set, with 3000 iterations per round, resulting in a dataset containing 183391 positive samples with the MaxSim greater than 0.6. Since there are only a few samples with different 2D but similar 3D structure (see Figure S4, Supporting Information), we extracted all samples with a MaxSim greater than 0.6 that are not in analogous region from the training dataset, with a total of 3356 361 samples. The two sets of positive samples were then merged, and then, negative samples were randomly selected from the sampled data with MaxSim less than or equal to 0.6 to complete the dataset, resulting in a total of 8000 000 samples. From the training set, 4501 samples with MaxSim greater than or equal to 0.75 and not located in analogous regions, were extracted to be used as an additional validation set. The validation set was sampled for two rounds, the test set for eight rounds, and the cross‐validation set for twenty rounds. The molecule pairs excluding any molecules present in the training set that had a MaxSim value greater than 0.6 and were not in analogous regions were excluded from the training set and used as an additional independent OOD test set.

### The Tokenizers and Training Regimens of the CrossEncoder

The tokenizer used in this work is a Byte‐Pair Encoding tokenizer,^[^ ^64^ ^]^ in which vocabularies were obtained by segmenting the SMILES of the compounds in our dataset. These tokenizers all include at least the basic elements that make up a pharmaceutical small molecule, such as C, N, O, S, P, c, n, o, s, F, Cl, Br, I, basic chemical bonds and isomer labels, such as, “= ”, “@”, “/”, “∖”, and “#”, as well as other special symbols, such as brackets and numbers.

We confirmed ELECTRA's superior performance in chemical languages in a previous study.^[^ ^65^ ^]^ In this work, we used the AutoGluon.^[^ ^25^ ^]^ framework to re‐train the ELECTRA‐Small.^[^ ^24^ ^]^ model (named SmELECTRA) using the hyperparameters as follows: learning rate was set to 0.001, weight decay was set to 0.001, learning rate schedule was set to cosine decay with 0.9, and the first 0.2 epoch was set to warmup steps. In these models, the CSS descriptor decoders are single linear layers. The training process was performed for a maximum of 20 epochs, and validated every 0.1 epochs. Up to three checkpoint models with the highest *R^2^* in the validation set were fused using the greedy soup.^[^ ^66^ ^]^ method and the final model was created.

### Graph‐Featurizers of the GeminiMol

A specific graph featurization approach was employed. The featurization process involves capturing diverse aspects of atoms and bonds within the molecular structure, which is crucial for accurately representing the chemical properties and interactions. For atom featurization, we utilized the concatenation featurizers provided by Deep Graph Library.^[^ ^67^ ^]^ (DGL) to combine multiple atom descriptors into node embedding of molecular graph. These descriptors include atom type, atom hybridization, atom formal charge, atom chiral tag, whether the atom is in a ring, and whether the atom is aromatic. Each descriptor provides valuable information about the atom's characteristics and its role in the molecular system. For bond featurization, we employed the canonical bond featurizers provided by DGL. This featurizers focuses on the bond type as the primary descriptor. The bond type indicates the nature of the chemical bond between two connected atoms and plays a crucial role in constructing the molecular graph.

### The Architecture Details and Training Regimens for GeminiMol

The overall architecture of GeminiMol is illustrated in Figure 1a. In the GeminiMol, the molecules are converted into molecular graphs and then subsequently encoded independently. In the GeminiMol model, the readout function was set to a multi‐layer perceptron that takes node features as input and computes their mean value. In the ablation experiments, dropping the layer number means decreasing the WLN layers to 2, while dropping the node features means decreasing the WLN node features to 1024 dimensions. In the GeminiMol model, the WLN graph consists of 4 layers, and the node dimensionality was set to 2048.

The projection head of the GeminiMol employed in our study was designed to transform the learned GeminiMol encoding into meaningful output predictions. The network consists of a series of fully connected layers with non‐linear activation functions and additional normalization to improve its learning ability. Two encoding vectors of query and reference molecules were inputted to the projection heads of CSS descriptors. The encoding vector first passes through a rectifier component, which expands the features to five times the dimension of the original encoding vector, batch normalization, and then reduces it to 1024 dimensions. Subsequently, the features gradually passed through linear layers of different sizes, reducing from 1024 dimensions to 128 dimensions and repeating this process three times. Finally, it outputs a value that was reset to the range of 0–1 through a sigmoid neuron in the projection head. The LeakyReLU^[^ ^68^ ^]^ activation function was added between all linear layers in the projection head. In the ablation experiment, a subset of models had the rectifier removed. For this subset, the encoding vector was directly reduced to 256 dimensions, and batch normalization was applied. Subsequently, the output was further reduced to 1 dimension after passing through LeakyReLU.^[^ ^68^ ^]^

For all GeminiMol models, the weighted Mean Squared Error Loss^[^ ^69^ ^]^ (MSELoss) function, the AdamW^[^ ^69^, ^70^ ^]^ optimizer with a learning rate of 0.0001 was used in training. In each training epoch, the training data was randomly rearranged and sampled 512 pairs per batch. We used two learning rate adjustment strategies during the training stage. First, the learning rate was gradually increased at the beginning of training. Second, cosine learning rate scheduling was used, which adjusts the learning rate according to the progress of the training steps to avoid falling into local optimal solutions. The starting learning rate of warmup was set to 0.1 times the original learning rate, which gradually increases to the original learning rate after 30 000 steps, and the minimum learning rate in the cosine learning rate schedule was 0.5 times the original learning rate, with a period of 10 000 steps. In addition, an early stopping strategy was used to determine whether the model converges by monitoring the performance on the validation set per 500 steps and terminating the training prematurely if there was no performance improvement in 50 validations iterations.

### Measuring Molecular Similarity

We defined three indices for measuring similarity between molecules: the 2D structures, the 3D conformational spaces, and the GeminiMol encoding vectors, respectively. In this work, we denote the query molecule A and the reference molecule B using ***A*** and ***B***. ***N*** was used to denote the number of molecular bonds and the size of the encoded vector. In the following Equation 1 for representing the MCS similarity, $N_{bonds}^{MCS}$ represents the number of bonds in the MCS, and thus, Similarity_*MCS*(A, B)_ represents the proportion of the structure overlapping between the two molecules to the structure of the smaller molecule. Matching of element types is not strictly required when calculating the MCS.

(1) $$\mathrm{Similarit}y_{MCS\left(A,B\right)}=\frac{N_{bonds}^{MCS}}{\min\left\{N_{bonds}^{A},N_{bonds}^{B}\right\}}$$

Besides, the CSS for pairs of molecules was represented by four raw CSS descriptors and advanced CSS descriptors in this work. The raw CSS descriptors were represented based on the minimum and maximum similarity of a pair of the global conformational space and a pair of the near‐native conformational space. For instance, Global_MAX represents the maximum similarity in the global conformational space, and so on. The advanced CSS descriptors are consisted of MaxSim, MaxDistance, MaxOverlap, MaxAggregation, CrossSim, CrossDistance, CrossOverlap, and CrossAggregation. Among them, MaxSim is the maximum value of all similarities, MaxDistance is the minimum value of these similarities, MaxOverlap is one‐half of the sum of MaxSim and MaxDistance, and MaxAggregation is one‐half of the sum of the maximum similarities under both strain energy thresholds. If “Max” is replaced by “Cross” in the names of the similarities in this work, this means half the value of the sum of the similarities of the molecules A to B and B to A. For cross‐similarities, the similarity of A to B and B to A are identical, while for biased similarity, they are different. For example, Tanimoto similarity is a cross‐similarity, while Tversky is a biased similarity.

Based on the obtained GeminiMol encoding vectors, calculating the similarity between pairs of molecules with the previously described vector similarity method can better describe the distances between molecules in latent space. This can be applied to various problems such as virtual screening, target identification, and scaffold hopping. For the encoding vector ***A*** of the query molecule and the encoding vector ***B*** of the reference molecule, we compute their cosine similarity and Pearson's correlation coefficient using following Equations (2), (3):

(2) $${\mathrm{Similarity}}_{\mathrm{Cosine}\left(A,B\right)}=\frac{A\cdot B}{\left(\left|A\right|\times \left|B\right|\right)}$$

(3) $${\mathrm{Similarity}}_{\mathrm{Pearson}\left(A,B\right)}=\frac{\mathrm{cov}\left(A,B\right)}{\left(\mathrm{stddev}\left(A\right)\times \mathrm{stddev}\left(B\right)\right)}$$

The GeminiMol encodes each molecule independently, allowing for a reliable calculation of the similarity between the encodings of two molecules, which in turn represents the similarity between the molecules themselves. This approach ensures a convincing representation of the conformational space profile of a single molecule.

### Creating Benchmark Datasets for Ligand‐Based Drug Discovery

In this work, we selected the DUD‐E^[^ ^26^ ^]^ and the LIT‐PCBA^[^ ^27^ ^]^ as the benchmark datasets for zero‐shot virtual screening. DUD‐E^[^ ^26^ ^]^ contained 102 targets and LIT‐PCBA^[^ ^27^ ^]^ contained 15 targets. The molecular fingerprints were used in the generation of the decoys in DUD‐E^[^ ^26^ ^]^ to avoid the production of “active” decoys. Therefore, thus the superior performance of the molecular fingerprints in DUD‐E^[^ ^26^ ^]^ is well deserved, as is that of the molecular docking and ligand similarity methods. In LIT‐PCBA,^[^ ^27^ ^]^ both the active and decoy molecules were obtained from high‐throughput screening, so the performance of all methods decreased significantly in this benchmark. For each target, DUD‐E^[^ ^26^ ^]^ provides one reference molecule and LIT‐PCBA^[^ ^27^ ^]^ provides multiple reference molecules. In the benchmark tests of LIT‐PCBA,^[^ ^27^ ^]^ there are multiple lead compounds for each target that can serve as reference molecules. The similarity was calculated of all reference molecules to the query molecules and select the highest similarity value as the final score for each query molecule.

In the scenario of evaluating virtual screening capabilities, DUD‐E^[^ ^26^ ^]^ is a much easier task compared to LIT‐PCBA,^[^ ^27^ ^]^ mainly due to the relatively fixed proportion of negative and positive compounds and the larger structural variations. However, it is very rare to come across such “standardized” tasks as DUD‐E^[^ ^26^ ^]^ in drug discovery. In contrast, there are often tasks as challenging as LIT‐PCBA,^[^ ^27^ ^]^ in particular, drug activity cliffs, complex mechanisms of action, and large differences in the proportion of active compounds in different tasks. Therefore, LIT‐PCBA^[^ ^27^ ^]^ is considered the main virtual screening benchmark test set in the study.

The data in BindingDB^[^ ^71^, ^72^ ^]^ was processed to create the zero‐shot target identification benchmark dataset (as shown in Figure S10, Supporting Information). In drug discovery, drug target identification is a challenging task, and the ligand similarity‐based methods are the dominant approach in this field. However, for molecules with apparently similar chemical structures, the computational method is not necessary (as illustrated in Figure S10a, Supporting Information). As shown in Figure S10b (Supporting Information), the benchmark dataset was produced by collecting decoy ligands with activity data entries greater than 300 and heavy atom numbers greater than or equal to 12 from BindingDB.^[^ ^71^, ^72^ ^]^ For each ligand, we removed targets with binding affinity greater than 10 µm and also removed all ligands with MCS similarity greater than 0.5 to mimic real‐world scenarios in which no similar structure compounds could be found from the bioactive database. Subsequently, the initial set of 176 decoy ligands was further refined by selecting only those ligands that targeted more than 70 receptors. This refined subset, consisting of 54 decoy ligands, was designated as the refined TIBD. The refined TIBD formulation comprises decoy molecules with diverse chemical structures, such as macrocyclic compounds like Ruboxistaurin, as well as Chlorpromazine (FDA‐approved drug) with a single ring system, and natural product Quercetin. Using Dendritic molecular fingerprint and Tanimoto similarity, the heatmaps display that there is low similarity among most molecules in TIBD, indicating a high diversity of decoy molecules (as shown in Figure S10c, Supporting Information). In the experiments, the decoy ligand was compared with all bioactive ligands in the decoy‐specificity library, and used the targets extracted from the matched ligands to analyze the ROC (as shown in Figure S10d, Supporting Information).

In addition, QSAR and ADMET benchmark datasets were elaborated corresponding to three different downstream tasks, including 21 single‐target datasets from PubChem BioAssays^[^ ^44^ ^]^ database and LIT‐PCBA,^[^ ^27^ ^]^ 73 cellular‐level datasets from the NCI/DTP, and 34 classification and 12 regression datasets of the ADMET properties from TDC^[^ ^43^ ^]^ and a drug addictive dataset from the literature.^[^ ^46^ ^]^ As mentioned above, datasets such as TDC^[^ ^43^ ^]^ and LIT‐PCBA^[^ ^27^ ^]^ provide an avenue to evaluate molecular representation models in real‐world application scenarios. Nevertheless, it is important to note that these datasets are predominantly influenced by target‐based drug design approaches, resulting in a dearth of comprehensive mechanism‐based phenotypic drug activity data. Most of the available QSAR datasets are derived from activity data specific to certain protein targets. The benchmark datasets incorporated a total of 73 cell line datasets from NCI/DTP to investigate the performance of molecular representation models in cellular‐level QSAR tasks.

In this study, all training, validation, and test sets were divided according to the molecular skeleton. For all datasets from TDC,^[^ ^43^ ^]^ we used the default scaffold split method to split the dataset (20% of data were held out for the test set). For other datasets from PubChem Bioassays, we used the Murcko Scaffold extractor provided by RDKit^[^ ^73^ ^]^ to identify the skeletons, the training and test sets were split according to the skeleton in a ratio of 7:3, where one‐tenth of the data in the training set was used as the validation set. For the asymmetric validation embedding (AVE) division provided by LIT‐PCBA,^[^ ^27^ ^]^ the training sets per targets were also divided into training and validation using the molecular skeleton in a ratio of 7:3.

In this study, a variety of statistical metrics were employed to assess the performance across various benchmark tasks. Among them, the Boltzmann‐enhanced discrimination of receiver operating characteristic (BEDROC) was used in the zero‐shot virtual screening and target identification. In this work, the α of BEDROC was set to 160.9, corresponding to EF_1%_.^[^ ^74^ ^]^ The receiver operating characteristic curve (AUROC) was used to binary classification problems, which contained seven QSAR datasets from LIT‐PCBA,^[^ ^27^ ^]^ and the high‐throughput assays data of CRF‐R2, CBFn‐RUNX1, NF‐*κ*B, Peg3, ARNT‐TAC3, A1 Apoptosis, uPA, Rango, Vif‐APOBEC3G, RGS12, HSD17B4, and HADH2 from PubChem BioAssays^[^ ^44^ ^]^ database, NCI cell lines, drug addictive, and the classification tasks of ADMET. The Spearman correlation coefficient was used in regression tasks of ADMET.

### Collecting Competitive Baseline Methods on these Benchmark Datasets

Over the past twenty years, various molecular representation methods have been developed to represent the features of small pharmaceutical molecules, including molecular fingerprints,^[^ ^31^, ^75^, ^76^ ^]^ pharmacophores,^[^ ^77^, ^78^, ^79^ ^]^ and molecular shapes,^[^ ^80^, ^81^, ^82^, ^83^ ^]^ which have continuously driven the technological development and the revolution of paradigm in drug discovery. The molecular fingerprint is the most popular molecular representation method, which has been continuously updated since its proposal in the last century and is still the most dominant method of molecular representation.^[^ ^84^ ^]^ Besides, the physics‐based methods, including pharmacophores and molecular shape‐based methods, such as AutoPH4,^[^ ^85^ ^]^ ROCS,^[^ ^86^ ^]^ and PhaseShape,^[^ ^21^ ^]^ enable simultaneous presentation of both the geometric shape and pharmacophoric features of molecules, allowing for numerical calculations to compare the similarity between molecules. These similarity computation methods are highly interpretable, can be applied to a wide range of chemical structure types, and have enabled practical applications during the past twenty years.

In this study, molecular fingerprints and PhaseShape were introduced as baseline methods to evaluate the performance of GeminiMol on zero‐shot tasks. To evaluate the performance of the models for virtual screening and target identification, the performance of 8 different molecular fingerprints (including ECFP4,^[^ ^31^ ^]^ ECFP6,^[^ ^31^ ^]^ FCFP4,^[^ ^31^ ^]^ FCFP6,^[^ ^31^ ^]^ MACCS,^[^ ^87^ ^]^ RDK,^[^ ^73^ ^]^ AtomPairs,^[^ ^47^ ^]^ and TopologicalTorsion^[^ ^32^ ^]^) and 8 different similarity metrics (including Tanimoto, Dice, Sokal, Cosine, Russel, Kulczynski, McConnaughey, and Tversky) in RDKit^[^ ^73^ ^]^ on the benchmark datasets were first benchmarked. For Tversky similarity, we tried to set α as 0.01, 0.50, and 0.99, respectively. The performance of different combinations of molecular fingerprints and similarity metrics varies significantly across different targets and tasks. As researchers in drug discovery, we often desire a method that exhibits stable performance rather than having to speculate which similarity metric would work best for a particular project before it begins. Therefore, in this study, we aim to test specific combinations of individual molecular fingerprints and individual similarity evaluation methods, with the hope of obtaining a method that demonstrates stable performance across various tasks and can make accurate predictions. For the reference molecule structures required by PhaseShape,^[^ ^21^ ^]^ we prepared them using the protein preparation module in the Schrödinger2021‐1 software package with default parameter settings that included assigning bond orders, adding hydrogens, and assigning partial charges.

For the downstream tasks of QSAR and ADMET, we compared GeminiMol, CrossEncoder, different molecular fingerprints, and CombineFP (combined by ECFP4,^[^ ^31^ ^]^ FCFP6,^[^ ^31^ ^]^ AtomPairs,^[^ ^47^ ^]^ and TopologicalTorsion^[^ ^32^ ^]^) using the same regimens of decoder selection and training, as described in the subsequent paragraphs. For the GeminiMol model, the hidden features extracted by the molecular encoder as the encoding vector for the molecule were utilized. For extracting molecular features using CrossEncoder, we utilized the API provided by AutoGluon^[^ ^25^ ^]^ to extract features for conformational space. In AutoGluon,^[^ ^25^ ^]^ the molecular SMILES was embedded separately and then concatenated together before it is input into the model, allowing for informational communication between molecules. To prevent the model from simply counting the same tokens in both molecular SMILESs to predict similarity, randomization was applied to the input SMILES to enhance the data. This randomization was also used in the feature extraction process. For the molecule from which we want to extract features, the canonical SMILES was set in place of the query molecule, while a random SMILES was generated and set in place of the reference molecule.

We have experimented with multiple decoder models for each downstream task, and select the best model as the final model. For each molecular fingerprint, CombineFP, GeminiMol, and CrossEncoder, we evaluated the same set of six downstream decoder models, including the CatBoost,^[^ ^88^ ^]^ three LightGBM models,^[^ ^64^ ^]^ and two neural network models. This approach mitigates the potential performance bias introduced by decoder selection. It is worth noting that, no hyperparameter tuning was used in downstream tasks. We employed AutoGluon^[^ ^89^ ^]^ to select the CatBoost,^[^ ^88^ ^]^ three LightGBM models,^[^ ^64^ ^]^ and first neural network without stacking, bagging, and hyperparameters trials. The neural network model consists of a linear layer that scales the features to 128 dimensions, then followed by a neural network composed of ReLU,^[^ ^90^ ^]^ dropout,^[^ ^91^ ^]^ and a linear layer, repeated three times. For classification problems, the output is projected to 2 dimensions and Softmax.^[^ ^92^ ^]^ For regression problems, the output is directly projected to 1D. The validation metric using AUROC for binary classification and Root Mean Squared Error (RMSE) for regression, respectively.

In addition, a PropDecoder network was integrated as a decoder in the QSAR and ADMET benchmarks, which has a similar architecture to the projection head in the GeminiMol network. Among all the decoder networks, only the PropDecoder supports fine‐tuning the encoder during the training process of downstream tasks. The selection of loss function and validation metrics for PropDecoder depends on the type of dataset. In terms of loss functions, for classification tasks, the loss was calculated by taking the mean of Binary Cross‐Entropy Loss.^[^ ^69^ ^]^ and MSELoss, for regression tasks, the loss was calculated by MSELoss. For datasets with a positive/negative sample ratio of more than 3 or less than one‐third, the area under Precision‐Recall curve.^[^ ^93^ ^]^ was used for validation metric, otherwise, the AUROC was employed. For regression tasks, the Spearman correlation coefficient was used for validation metric. Similar to the training process of the GeminiMol network, the AdamW.^[^ ^69^, ^70^ ^]^ optimizer was utilized during the training of PropDecoder. The complexity of the model, learning rate, and number of epochs were dynamically adjusted to adapt to varying dataset sizes. For larger datasets, a larger learning rate, more complex network architecture, larger batch sizes, and fewer epochs were employed. Conversely, for smaller datasets, a smaller learning rate, simpler network architecture, smaller batch sizes, and more epochs were utilized. The code for training PropDecoder was also available in our source code repository (https://github.com/Wang‐Lin‐boop/GeminiMol).

Besides, Uni‐Mol was also compared,^[^ ^7^ ^]^ representing deep learning molecular representation models, and FP‐GNN,^[^ ^48^ ^]^ representing the integrated approach based on multiple molecular representations. For Uni‐Mol,^[^ ^7^ ^]^ the data set splitting followed the same scheme as the molecular fingerprints and the models, the validation metrics, and all hyperparameters were left at their default settings. For FP‐GNN,^[^ ^48^ ^]^ the data set splitting, validation metrics, and all hyperparameters are left at their default settings.

### Statistical Analysis

In this study, all statistical data in the figures were presented without transformation, normalization, or removal of outliers. In Figure 4, the top and bottom of the box plot represent the first and third quartiles, respectively. The mean was indicated by a horizontal line, and the median is represented by an empty circle. The sample sizes for all statistical analyses can be found in the figure or its caption. All statistical graphs were generated using OriginPro 2021 software.

### Data Availability

All related data are freely available from public sources. The training data and benchmark datasets in this work were stored in the Zenodo repository (CSS and Benchmark Datasets of GeminiMol, https://doi.org/10.5281/zenodo.10273479), pre‐trained models were stored in Hugging Face (GeminiMol, https://doi.org/10.57967/hf/2825, Link: https://huggingface.co/AlphaMWang/GeminiMol). The BioAssay ID and descriptions of the dataset can be found in Table S1 (Supporting Information), while the statistical metrics for virtual screening and target identification benchmark tests were presented in Table S2 (Supporting Information). Additionally, the statistical metrics for QSAR and ADMET benchmark tests can be found in Table S3 (Supporting Information).

### Code Availability

Source code for the GeminiMol model, trained weights, training, and inference script are available under an academic free license at our GitHub repository (https://github.com/Wang‐Lin‐boop/GeminiMol), Neural networks were developed with PyTorch (pytorch.org), AutoGluon (auto.gluon.ai), and DGL‐Life (lifesci.dgl.ai).

## Conflict of Interest

The authors declare no conflict of interest.

## Supporting information

Supporting Information

Supplemental Table 1

Supplemental Table 2

Supplemental Table 3

## Data Availability

The data that support the findings of this study are openly available in Zenodo at [https://doi.org/10.5281/zenodo.10273479], reference number 10273479.
