# Supplementary material for: Conformational Space Profiling Enhances Generic Molecular Representation for AI‐Powered Ligand‐Based Drug Discovery
Source: Adv Sci (Weinh). 2024 Aug 29;11(40):2403998. doi: 10.1002/advs.202403998 (PMC11516098; doi:10.1002/advs.202403998)
Supplement: Supplementary file 1 — Supporting Information [file ADVS-11-2403998-s001.docx]

Supplementary Information

Conformational Space Profiling Enhances Generic Molecular Representation for AI-powered Ligand-based Drug Discovery

Lin Wang, Shihang Wang, Hao Yang, Shiwei Li, Xinyu Wang, Yongqi Zhou, Siyuan Tian, Lu Liu, Fang Bai*

**Supplementary Table**

Table S1. | General description of QSAR benchmark datasets used in this work.

Table S2. | Statistical data of all molecular fingerprints, CrossEncoder, and GeminiMol models in virtual screening and target identification benchmarks.

Table S3. | Statistical data of all molecular fingerprints, CrossEncoder, and GeminiMol models in QSAR and ADMET benchmark tasks.

**Supplementary Figures**


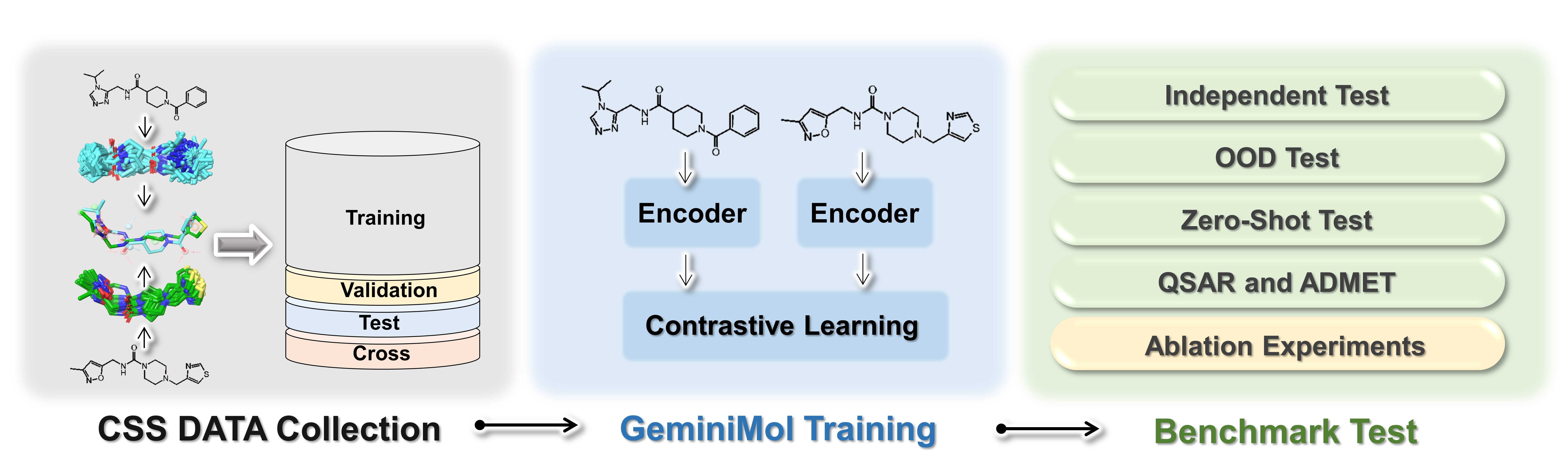


**Figure S1. The overall workflow in this work.** In this schematic diagram, the gray blocks represent physics-based data collection and the proof-of-concept study, the blue blocks represent model training, and the green blocks represent benchmark tests with different downstream tasks.


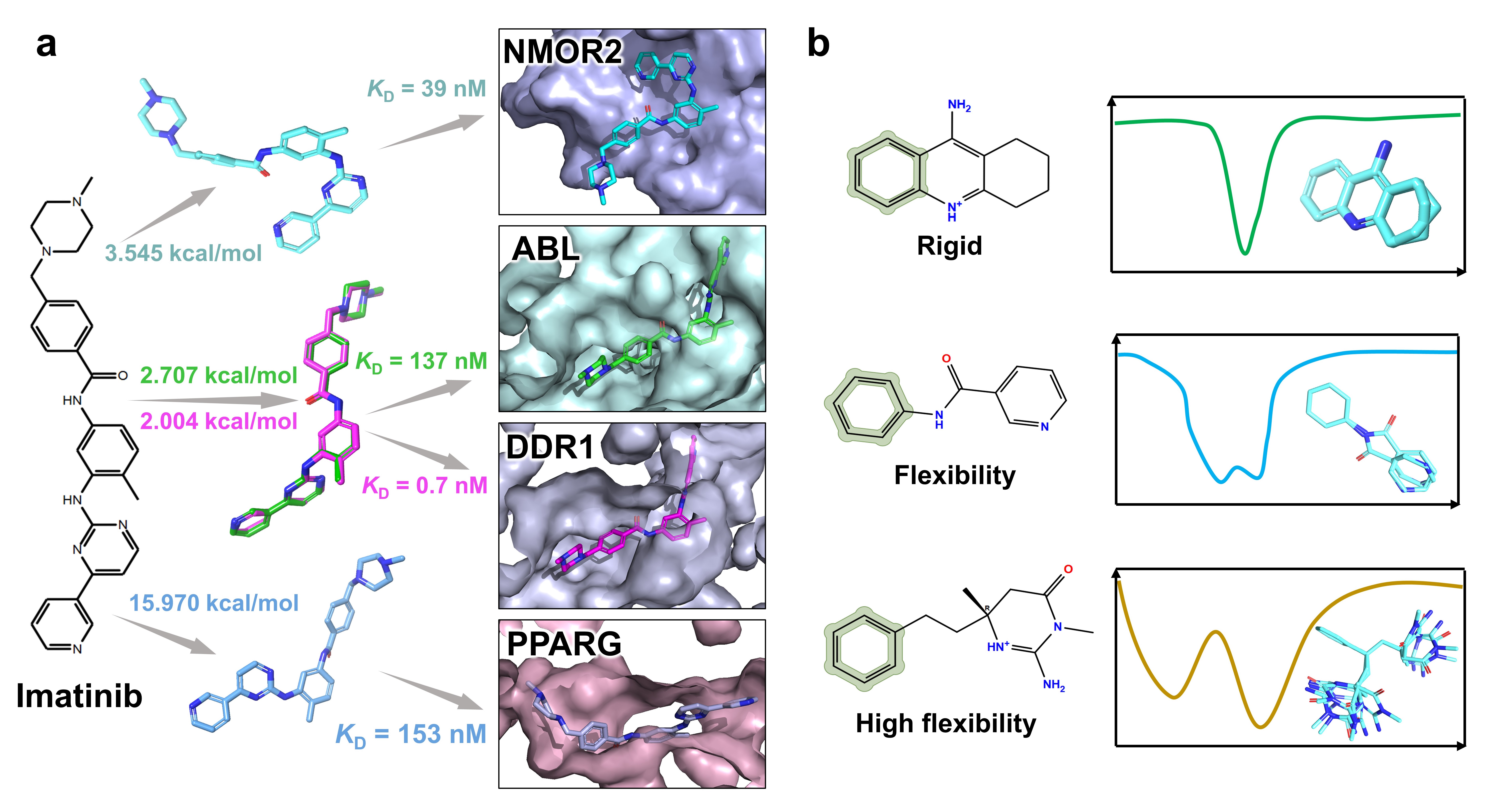


**Figure S2. The significance of conformational space information for the biological activity of drug molecules. a,** Example of imatinib, which binds to four different targets with three different conformations. The energies in kcal/mol in this figure represented strain energy. The PDB IDs corresponding to the ligand structures from top to bottom are 3FW1, 3PYY, 4BKJ, and 6KTN. **b,** As the number of rotatable bonds increases, the conformational space of a small molecule becomes more complex.


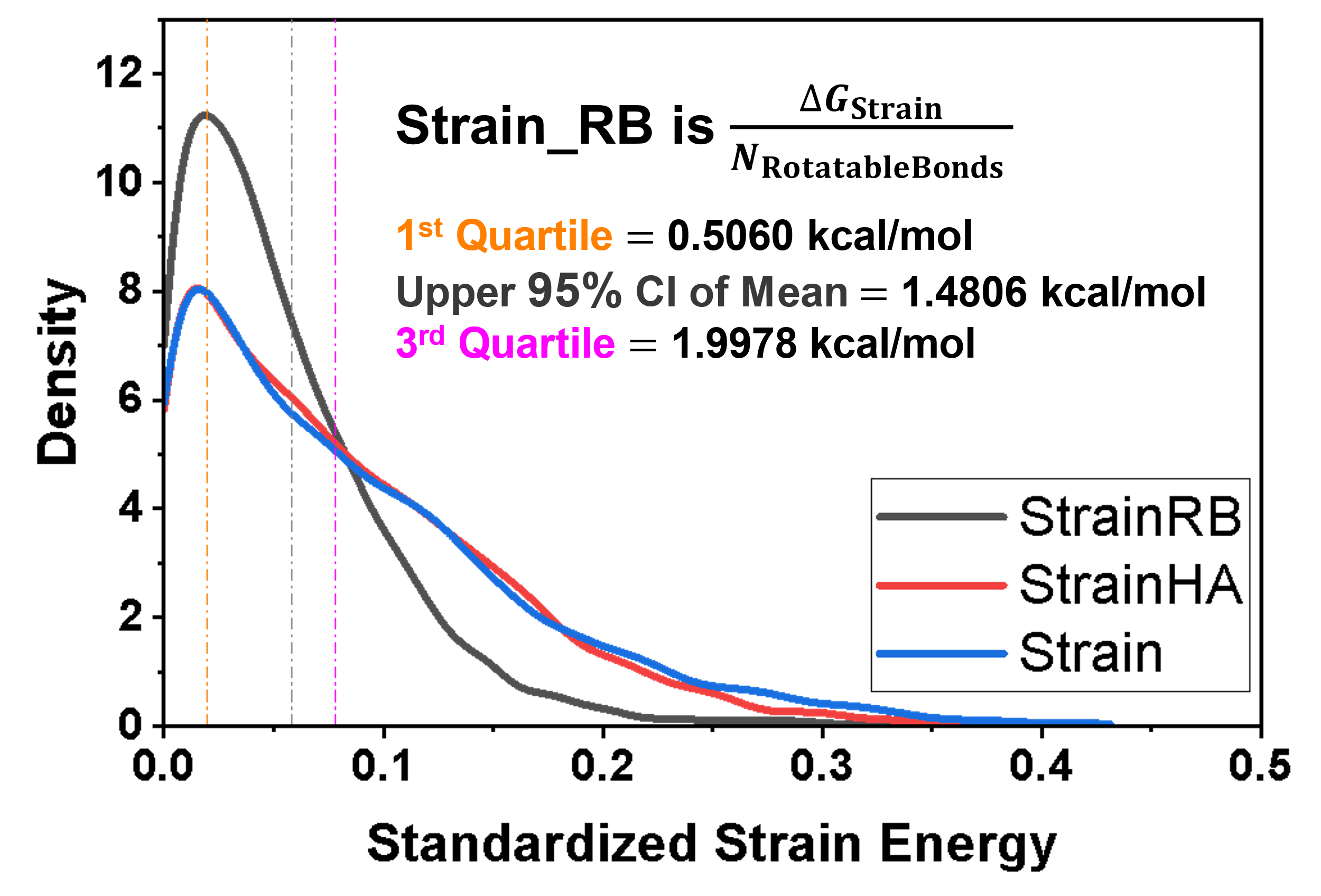


**Figure S3. Investigating the impact of molecular size on the strain energy distribution of molecular conformations in the PDB.** This density plot obtained by standardizing the ratio between strain energy and measurements of different molecular sizes, reveals the relationship between molecular size factors and strain energy. The strain energy factored by the number of rotatable bonds exhibits the most optimal distribution curve. RB, rotatable bonds, HA, heavy atoms.


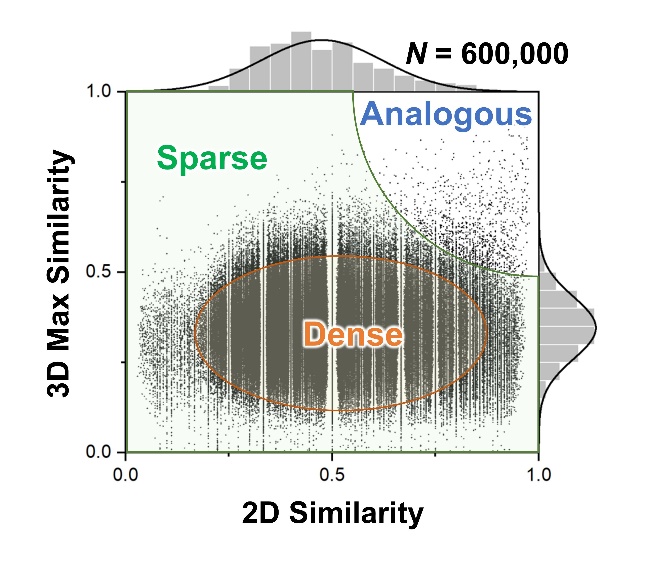


**Figure S4. Relationship between the MaxSim and MCS similarity scores for the molecule pairs.** The plot shows an ellipse defining a region with a high density of molecule pairs, and a sector to define a region with high MCS similarity and MaxSim values. During the sampling of the, different weights are assigned to these two regions together with the remaining regions to achieve a balanced data sampling.


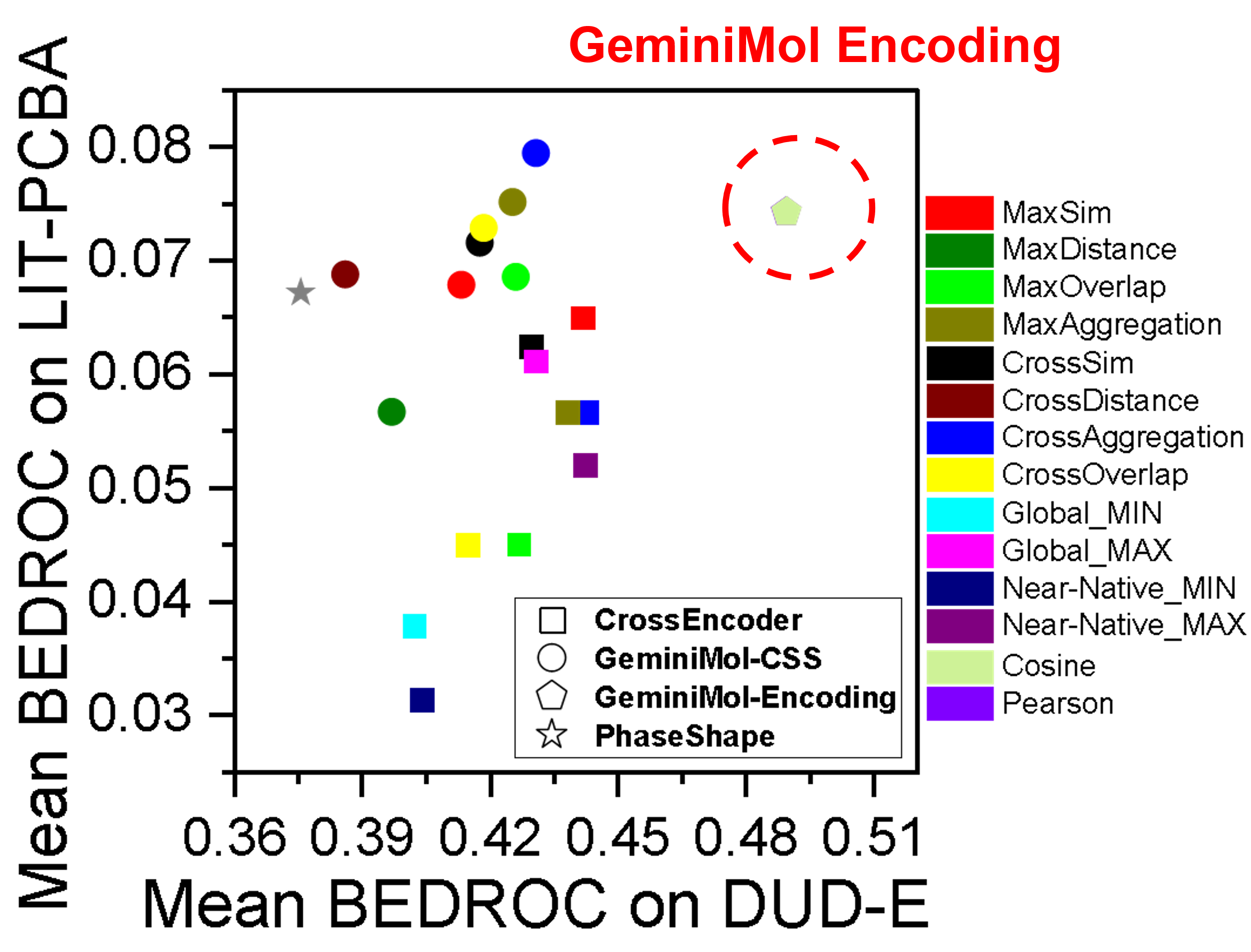


**Figure S5. The virtual screening performance comparison of PhaseShape and our models.** The various predicted CSS descriptors were represented by different colors. For instance, Global_MAX represents the maximum similarity in the global conformational space, and so on. Among them, MaxSim represents the maximum value of all similarities, MaxDistance represents the minimum similarity, MaxOverlap represents one-half of the sum of MaxSim and MaxDistance, and MaxAggregation represents one-half of the sum of the maximum similarities under both strain energy thresholds. If “Max” is replaced by “Cross” in the names of the similarities in this work, this means half the value of the sum of the similarities of the molecules A to B and B to A. The portion highlighted in red dashed lines represents the similarities of GeminiMol encoding vector, including Pearson, Cosine, Manhattan, and RMSE.


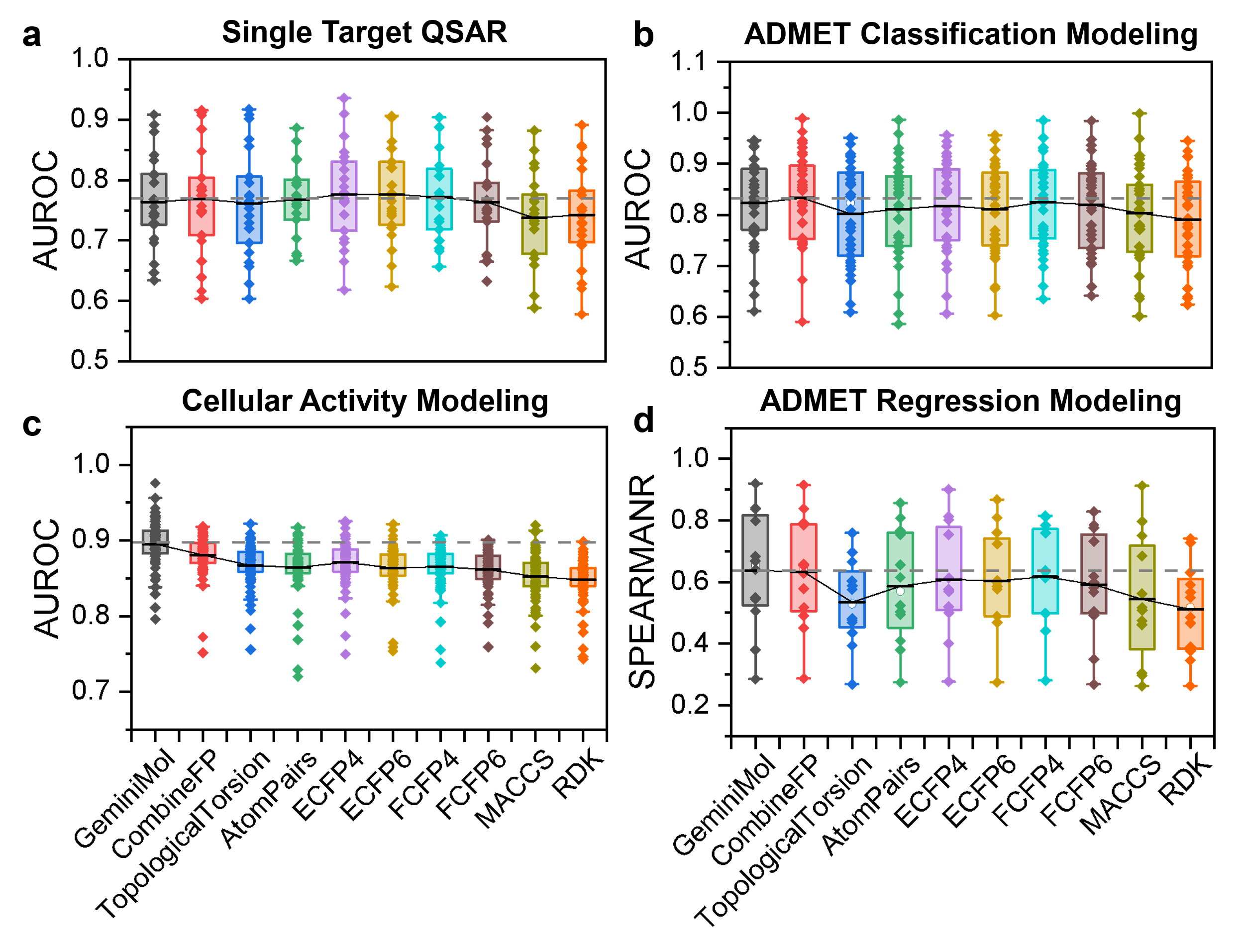


**Figure S6. The performance of the GeminiMol model and molecular fingerprints across four different downstream tasks. a,** Target-based QSAR. The data for this task was sourced from the PubChem BioAssay database and LIT-PCBA. All activity data exhibit dose-response relationships and are associated with specific target proteins. **b,** ADMET classification task. **c,** Phenotype-based QSAR task. This task focuses on QSAR modeling using data from 73 cancer cell lines obtained from NCI/DTP. **d,** ADMET regression task. The dashed line indicates the performance of the GeminiMol-MOD model. GeminiMol-MOD is a modified version of the GeminiMol model, with the main distinction being that GeminiMol-MOD can extract more information from molecular graphs of the same size.


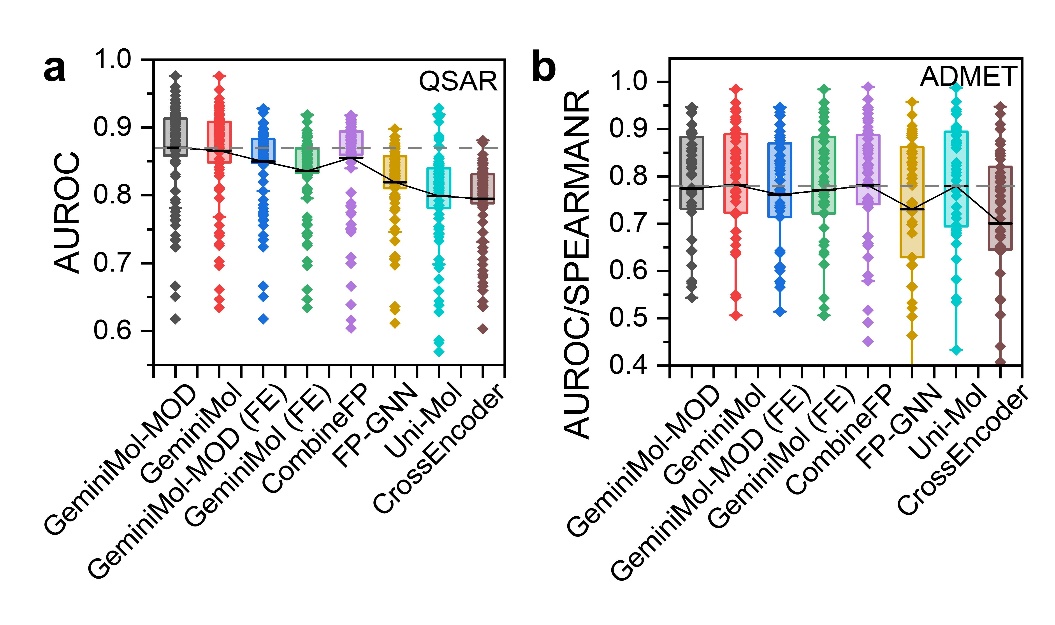


**Figure S7. The impact of fixed the encoder on the performance of GeminiMol for QSAR and ADMET tasks. a,** The QSAR performance comparison of freezing GeminiMol encoder with other methods. **b,** The ADMET performance comparison of freezing GeminiMol encoder with other methods.


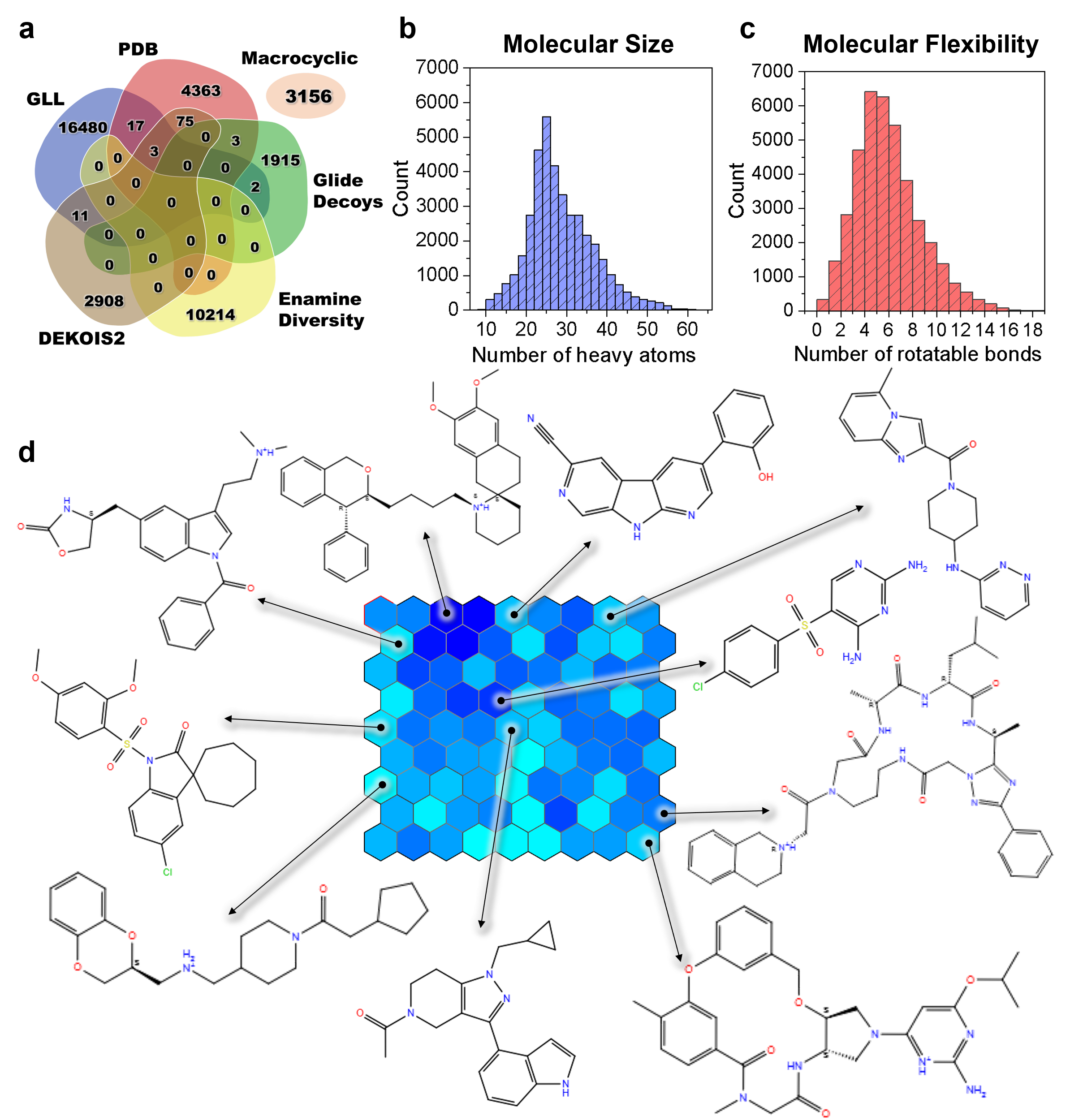


**Figure S8. The introduction of the small-scale molecular dataset.** **a,** The source and overlaps between different sources were marked numerically in a Venn diagram. **b,** The distribution of molecular size in our molecular dataset. **c,** The distribution of rotatable bond in our molecular dataset. **d,** The SOM for visualizing the distribution of molecular structures in the compound dataset used in this work. The shown molecules were randomly selected from the cells. The SOM was created by 96 most informative bits of the pairwise fingerprint, and color gradient within cell population 114 - 1,000, the max population is 1,673.

**
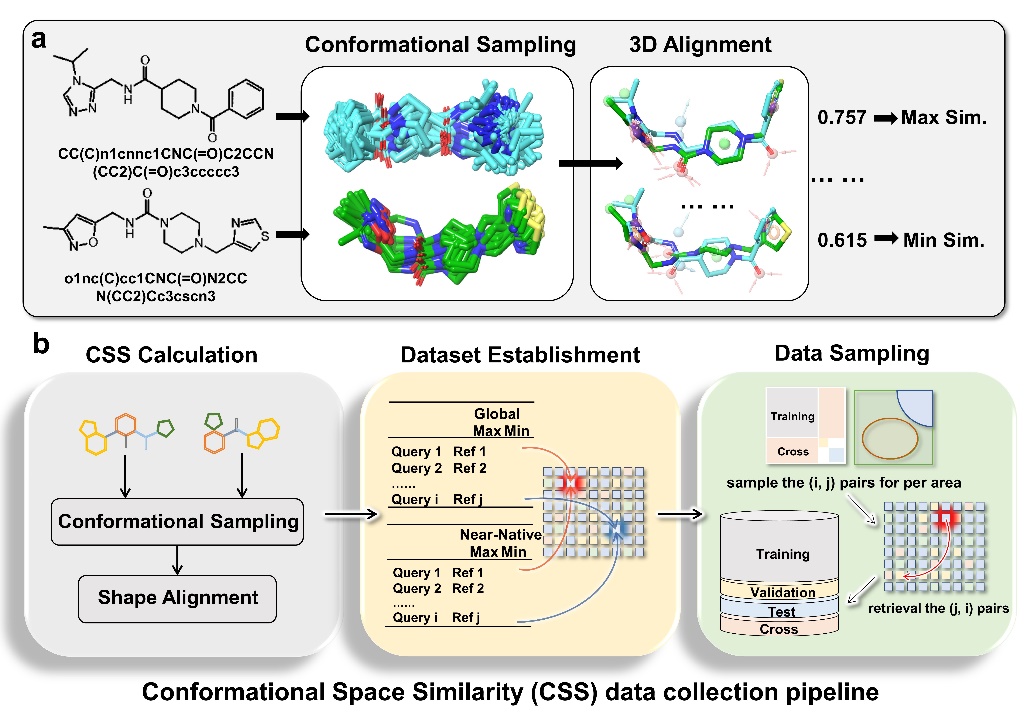
**

**Figure S9. The workflow of data acquisition of CSS descriptors. a,** The flowchart illustrates the process of comparing the molecular CSS between two molecules. **b,** Upon calculating the CSS, the obtained CSS descriptors under different conditions are then inserted into a single matrix. Subsequently, symmetrically extracted CSS descriptors from the matrix are employed for the training, validation, and testing of the model.


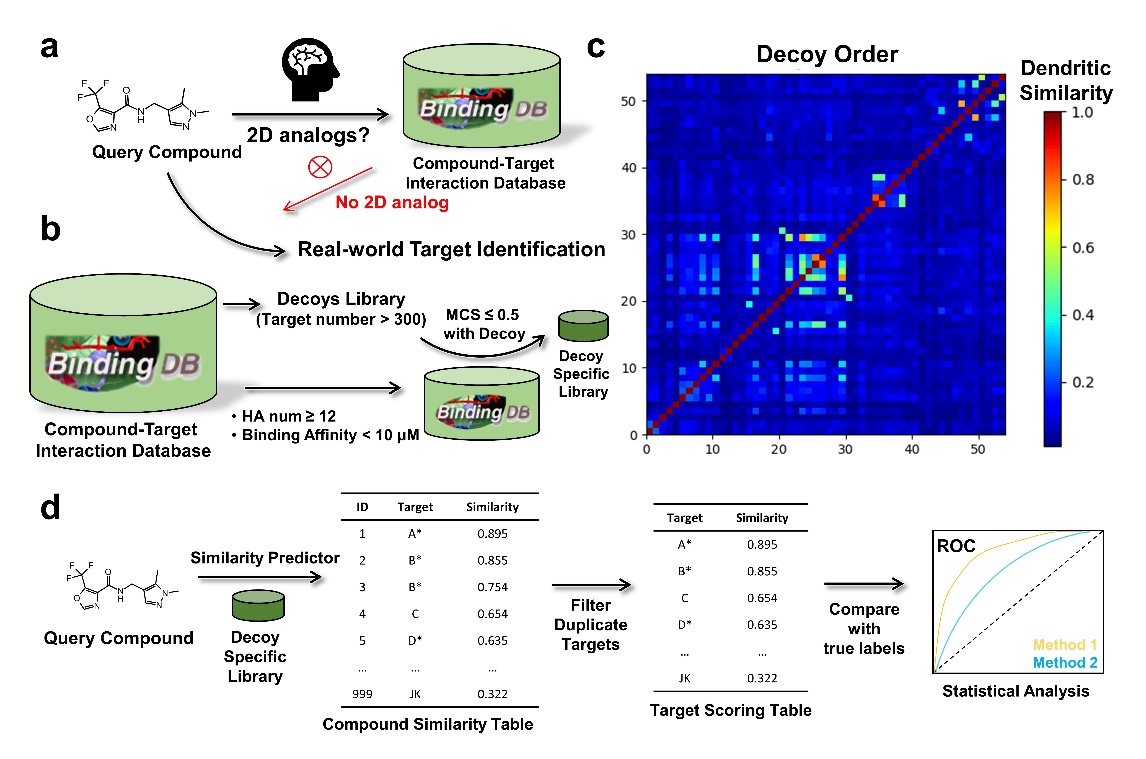


**Figure S10. Motivation, process and test methods for creating a benchmark target identification dataset.** **a**, For target identification datasets, query molecules with 2D analogues are easier to study. Therefore, a database thatdoes not contain 2D analogues of query molecules can better reflect the real-world scenarios in target identification tasks. **b,** Method for creating the benchmark test dataset. **c,** Heatmap of fingerprint similarity of decoys. The similarity metric is Tanimoto. **d,** Proposed evaluation scheme for target identification methods in this study.
